# MELOE-1 Antigen Contains Multiple HLA Class II T Cell Epitopes Recognized by Th1 CD4+ T Cells from Melanoma Patients

**DOI:** 10.1371/journal.pone.0051716

**Published:** 2012-12-20

**Authors:** Mathilde Bobinet, Virginie Vignard, Anne Rogel, Amir Khammari, Brigitte Dreno, Francois Lang, Nathalie Labarriere

**Affiliations:** 1 Inserm, U892, Nantes, France; 2 Université de Nantes, Nantes, France; 3 CNRS, UMR 6299, Nantes, France; 4 Centre Hospitalier Universitaire (CHU) Nantes, Nantes, France; Massachusetts General Hospital, United States of America

## Abstract

MELOE-1 is an overexpressed melanoma antigen containing a HLA-A2 restricted epitope, involved in melanoma immunosurveillance of patients adoptively transferred with tumour infiltrating lymphocytes (TIL). The use of the full-length antigen (46 aa) for anti-melanoma vaccination could be considered, subject to the presence of Th epitopes all along MELOE-1 sequence. Thus, in this study we evaluated *in vitro* the immunoprevalence of the different regions of MELOE-1 (i.e. their ability to induce CD4 T cell responses *in vitro* from PBMC). Stimulation of PBMC from healthy subjects with MELOE-1 induced the amplification of CD4 T cells specific for various regions of the protein in multiple HLA contexts, for each tested donor. We confirmed these results in a panel of melanoma patients, and documented that MELOE-1 specific CD4 T cells, were mainly Th1 cells, presumably favourable to the amplification of CD8 specific T cells. Using autologous DC, we further showed that these class II epitopes could be naturally processed from MELOE-1 whole protein and identified minimal epitopes derived from each region of MELOE-1, and presented in four distinct HLA contexts. In conclusion, vaccination with MELOE-1 whole polypeptide should induce specific Th1 CD4 responses in a majority of melanoma patients, stimulating the amplification of CD8 effector cells, reactive against melanoma cells.

## Introduction

In antitumor immune responses, CTL have been identified as the most powerful effector cells [1]. As a consequence, most previous anti-cancer vaccines use class I HLA-restricted peptides derived from tumour antigens in order to stimulate CTL responses. However, the clinical impact of such peptide-based cancer vaccines remains still modest, even if a recent gp100-derived peptide vaccination was shown to increase patient survival in melanoma [2], [3]. In addition to a variety of immune suppressive mechanisms originating from the tumour itself, suboptimal design of vaccines used so far may explain this failure. In particular, short epitopic peptides, could induce vanishing CTL responses or tolerance towards targeted antigens [4], [5]. In the meanwhile, CD4 helper T cells have gained interest in anti-tumour immunity and immunotherapy [6]. Indeed, tumour-reactive CD4^+^ T helper 1 cells (Th1) produce several cytokines (such as IFN-fγ, TNF-α and IL-2) essential for the induction of cell-mediated immunity against tumours [7]. One widely accepted model demonstrates the ability of CD4^+^ T cells to ‘license’ dendritic cells (DCs) for efficient CD8^+^ T cell priming through the interaction of costimulatory receptors [8], [9]. The cytokines secreted by CD4^+^ Th1 cells also exert direct antitumor and antiangiogenic effects [10]. Furthermore, it has been demonstrated in a mouse model that only tumour-reactive CD4^+^ T cells have been found to ensure efficient effector CTLs recruitment at the tumour site [11]. From a clinical standpoint, a high density of tumour-infiltrating CD4^+^ Th1 cells has been recently shown as a good prognostic marker in colorectal cancer patients emphasizing the role of these cells in cancer immunosurveillance [12]. In melanoma, tumour-reactive CD4 T cells have also been associated with a good clinical outcome [13], and more recently the same group showed that tumour specific CD4 T cells were present in at least 20% of metastatic melanomas, and suggested that the infusion of TIL (Tumour Infiltrating Lymphocytes) populations containing CD4 specific T cells could enhance the efficacy of adoptive cell therapy [14]. In the same line of thought, it has been demonstrated in a melanoma patient that the adoptive cell transfer of CD4 T cells specific for NYESO-1 antigen induced durable clinical remission and led to endogenous responses against non-targeted tumour antigens, suggesting the stimulation of immune responses by transferred CD4 T cells [15].

In the field of peptide vaccination, it has been documented twenty years ago, in a mouse model, that the generation of a strong CD8 response against a LCMV-derived peptide depended on the presence of CD4 helper T cells [16]. These results have been more recently confirmed in a clinical setting by the use of synthetic long peptides (SLP) in colorectal cancer, using P53 derived SLP [17], in vulvar intraepithelial neoplasia [18] and cervical cancer patients [19] using HPV16-derived SLP. In the case of vulvar neoplasia, clinical responses appeared to be correlated with the induction of strong HPV16 specific immune responses [18]. SLPs containing immunogenic CD8 and CD4 tumour epitopes are therefore attractive tools to implement therapeutic cancer vaccine.

One of the main issues in the field of SLP vaccination in solid tumours is to identify immunogenic long peptides derived from relevant tumour associated antigens. Target antigens should be widely expressed in tumour cells, and able to induce robust CD8 and CD4 anti-tumour T cell responses. In melanoma, the Melan-A antigen fulfils these requirements and we recently reported the efficiency of a Melan-A modified SLP, to cross-prime human tumour-reactive T cells. [20]. Another attractive target for melanoma vaccination would be the MELOE-1 antigen (46 aa), overexpressed in melanoma. Indeed, we previously reported that the infusion of TIL specific for this antigen was associated with a prolonged relapse-free survival for HLA-A2 melanoma patients who received TIL therapy [21]. Furthermore, we documented the presence of a large and tumour reactive CD8 T cell repertoire in HLA-A2 melanoma patients [22] and the presence of two class II epitopes in the vicinity of the class I epitope, located at the C-terminal end of the polypeptide [23].

In this study, our objective was to define the ability of this promising melanoma antigen to induce CD4 specific T cells *in vitro*, from healthy subjects and melanoma patients. This ability, called “immunoprevalence” can be defined as the frequency of responders [24]. This property is a requirement for the design a therapeutic vaccine using full-length MELOE-1 antigen or a mixture of selected long peptides for melanoma patient’s vaccination. The proof of concept will be achieved by the characterization of class II epitopes located all along the MELOE-1 sequence and of the Th profile of CD4 T cell specific lymphocytes.

## Materials and Methods

### Cells

Blood samples from healthy subjects and melanoma patients were respectively obtained from the “Etablissement Français du Sang”, Nantes, France and from the department of Onco-Dermatology, Nantes Hospital, France. Patients were stage III to IV melanoma patients. Melanoma and B-EBV cell-lines were maintained in RPMI 1640 (GIBCO) containing 10% FCS (PAA). Lymphocytes were grown in RPMI 1640 8% human serum (HS) with 50 or 150 IU/ml of recombinant IL-2 (Chiron, Proleukin® Aldesleukin) and 2 nM of L-Glutamine (GIBCO). For experiments using DC, RPMI supplemented with 20 mg/mL of human albumin (LFB, Viabelex 200 mg/mL) was used to avoid peptide degradation by serum proteases.

### Ethics Statement

Written consents were obtained from all patients. This study was approved by the local ethic commmitee of Nantes hospital (GNDES), and registered under the CNIL number « 1278197 ».

### Reagents

**Figure 1:**
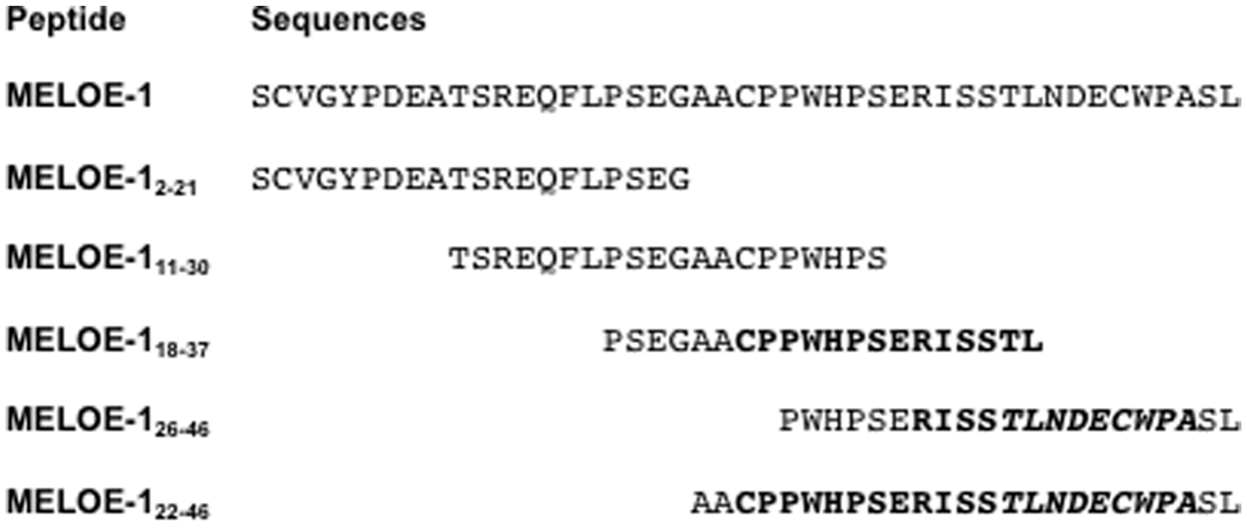
MELOE-1 and MELOE-1 derived peptide sequences. All the peptides were purchased from Millegen company (France), with a purity >85%. In bold are indicated the DR-11 (CPPWHPSERISSTL) and the DQ-6 (RISSTLNDECWPA) overlapping epitopes already described, and in italics is indicated the HLA-A2 restricted class I epitope (TLNDECWPA).

Antibodies (CD4-PE, CD27-PE, CD28-PE, CD45RO-PE, CD45RA-PE, CD14-PE, GMCSF-PE, IL-4-APC, IL-4-PE, IL-2-PE, IL-5-PE, IL-13-PE) were purchased from BD Biosciences or from Miltenyi Biotec (CD3-PE, CD8-FITC, CD4-APC, IFN-γ-FITC, TNF-α-APC, TNF-α-PE, IL10-PE). Purified cytokines (GMCSF, IL-4, TNF-α) were purchased from CellGenix. The different peptides (Millegen, purity >85%) used in this study are described in Figure 1. HLA-A*0201/MELOE-1_36–44_ monomers were generated by the recombinant protein facility of our institute (SFR Santé).

### Stimulation of MELOE-1 Specific T cells

PBMC from healthy donors or melanoma patients (2.10^5^ cells/well) were cultured for 14 days with 10 µM of MELOE-1 whole antigen (46 aa) in RPMI medium supplemented with 8% HS, 50 IU/ml of rIL-2 and L-Glutamine, in 96–well multiplates. Microcultures were then restimulated individually with 10 µM of each overlapping peptide (MELOE-1_2–21_, MELOE-1_11–30_, MELOE-1_18–37_, MELOE-1_26–46_ or MELOE-1_22–46_ for melanoma patients) in the presence of 10 µg/mL brefeldin A (BFA, Sigma Aldrich), for 5 h and the percentage of CD4^+^ specific T cells was assessed by TNF-α, IFN-γ or IL-4 intracellular staining. A negative control without peptide was included in all experiments.

### Dendritic Cells Generation and Loading

Monocytes were purified from PBMC of healthy donors by a CD14-enrichment kit, according the recommendations of the supplier (STEMCELL). Immature DC (iDC) were generated by culturing monocytes in RPMI supplemented with 20 mg/mL of human albumin, 1000 IU/mL of GMCSF and 200 IU/mL of IL-4 for 5 days. Then, iDC were pulsed with the whole MELOE-1 (1 µM) protein or the modified Melan-A_16–40_ A27L as negative control (1 µM) and matured with 20 ng/mL of TNF-α and 50 µg/mL of PolyI:C (Sigma Aldrich) for 4 h at 37°C. Finally they were fixed for 1 min with PBS/0.015% glutaraldehyde (Sigma Aldrich). Alternatively, iDC were first matured, fixed and then pulsed with antigens at the same concentration. MELOE-1 specific CD4+ T cell clones were then stimulated by autologous MELOE-1 loaded DC at a 1∶1 ratio, and analyzed by intracellular staining, with anti-CD3/TNF-α double labelling.

### Cytokine Intracellular Staining

Lymphocytes were stimulated for 5 h in the presence of BFA (10 µg/mL) either with peptide alone (10 µM) in an autopresentation assay or with B-EBV or HLA-class II expressing melanoma cells pulsed 2 h with the cognate peptide, at an effector: target ratio of 1∶2. In some experiments, blocking mAb against HLA-DP (clone B7.21 from Dr Charron, UMR940, Paris), HLA-DQ (clone SPVL3, Beckman Coulter) or HLA-DR (clone L243, BD Biosciences) were added at a concentration of 12.5 µg/ml. Cells were then stained with APC-conjugated anti-CD4 mAb, fixed with 4% paraformaldehyde (Euromedex), labelled with PE-conjugated anti-cytokine mAb and analyzed by flow cytometry.

### T cell Cloning and TCR Characterization

Polyclonal cultures containing specific CD4+ T cells were cloned by limiting dilution as previously described [25]. After 2 weeks, the peptide specificity of each clone was assessed by a TNF production assay. For TCR sequencing, RNA from 5.10^6^ T cell clones was extracted with NucleoSpin RNA II (Macherey Nagel), according to the supplier’s instructions. Reverse transcriptions, PCR amplifications and sequencing were performed as described [26]. We used the TCR nomenclature established by Arden *et al*. [27].

## Results

### Frequency and Distribution of MELOE-1 Specific CD4 Responses in Healthy Donor’s PBMC Stimulated with MELOE-1 Antigen

**Figure 2:**
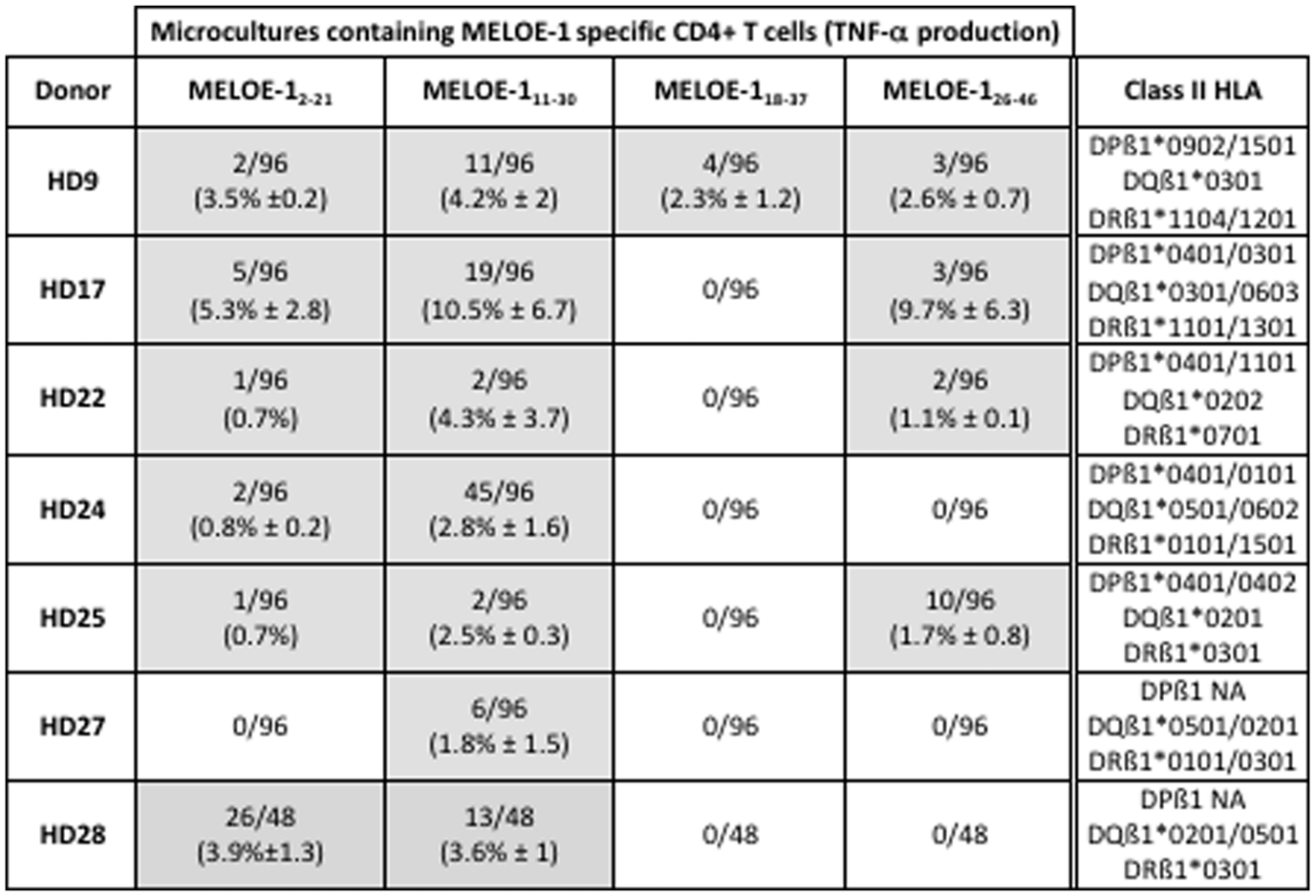
Assessment of MELOE-1 CD4 T cell responses in PBMC from healthy donors. PBMC from healthy donors were stimulated with 10 µM of MELOE-1. After 14 days, the presence of CD4 T cells specific for the different regions of MELOE-1 was assessed by re-stimulating cells with MELOE-1_2–21_, MELOE-1_11–30_, MELOE-1_18–37_ and MELOE-1_26–46_ peptides, followed by CD4/TNF-α double staining and flow cytometry analysis. Between brackets is indicated the mean % of TNF-α producing CD4 T cells, in positive microcultures. NA: not available.

**Figure 3:**
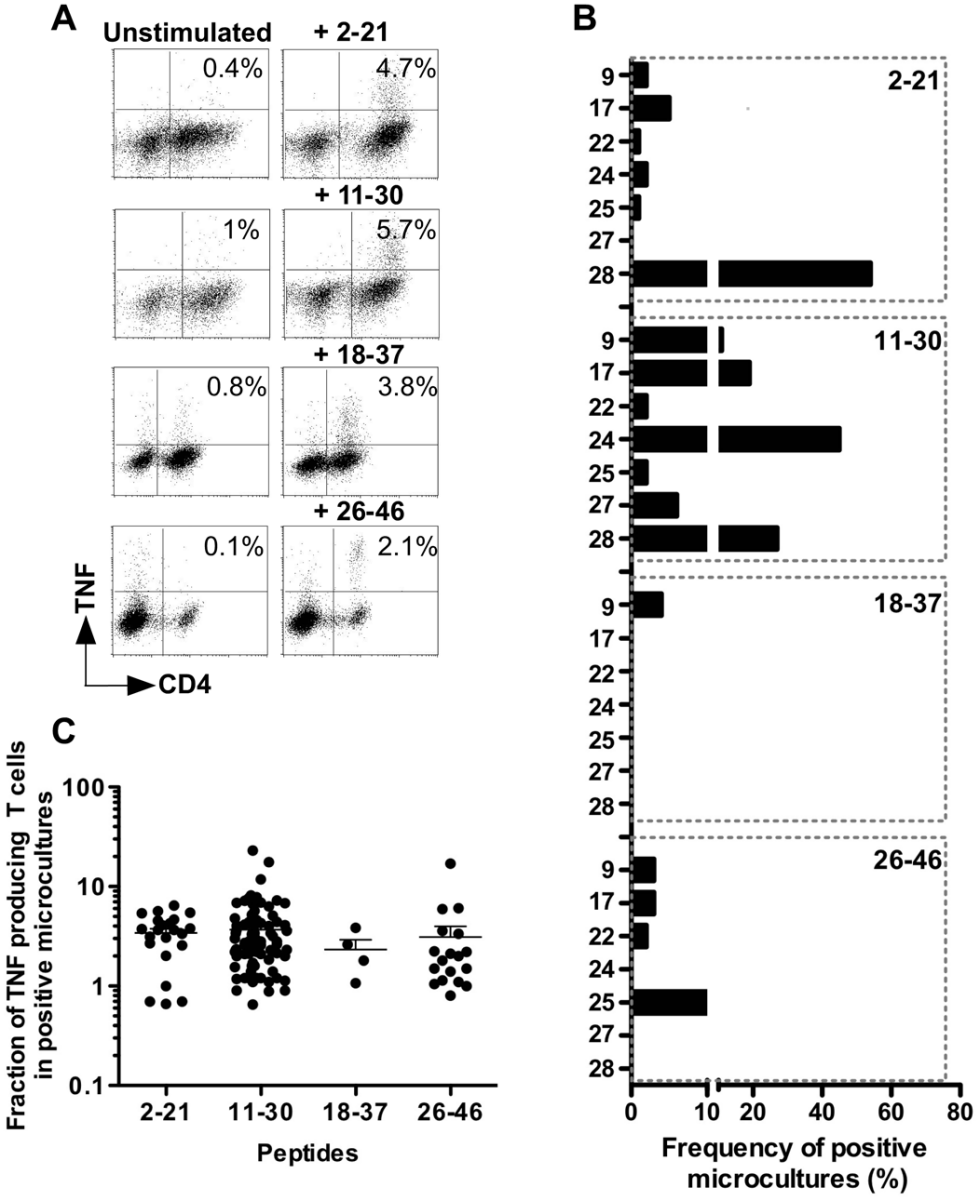
MELOE-1 specific responses in healthy donors. (A) Dot plot illustrating peptide-specific TNF responses, in MELOE-1 stimulated microcultures from HD9 and HD28. Fourteen days after PBMC stimulation with MELOE-1 whole polypeptide (2–46), microcultures were re-stimulated or not with each indicated peptide during 5 h. Reactivity was then assessed by double staining TNFα/CD4. Percentages indicate the fraction of TNF producing T cells, among CD4 T cells. (B) Frequency of microcultures containing CD4 T cells specific for MELOE-1 peptides. A microculture was considered as positive when the fraction of TNF producing T cells was 2-fold higher upon peptide stimulation than in unstimulated cultures. (C) Percentages of TNF-α producing CD4 T cells upon peptide-stimulation, in positive microcultures from all donors.

Our purpose was to look for the existence of class II helper epitopes all along the MELOE-1 sequence, in order to document the immunoprevalence of the different regions of this melanoma antigen. We stimulated 2.10^7^ PBMC from seven healthy donors with MELOE-1 whole antigen and tested, after a 14-day culture period, the presence of CD4 T cells specific for each region of the protein. Microcultures were screened for TNF-α production by CD4+ T cells, after restimulation with four MELOE-1 derived overlapping peptides (Figure 1), in an autopresentation assay. As shown in Figure 2, all donors exhibited CD4 responses against at least 1 out of 4 overlapping peptides. Considering that 2×10^7^ PBMC were stimulated from each donor, and that we detected between 5 to 47 microcultures containing CD4 T cells specific for the different regions of MELOE-1, we could roughly estimate the frequency of MELOE-1 specific CD4 precursors from 6×10^−7^ to up to 6×10^−6^ among CD4 T cells from healthy donors. Figure 3A shows representative examples of peptide-specific responses in microcultures from HD9 and HD28 (right panel), and of the corresponding TNF background in unstimulated microcultures (left panel).

As illustrated by Figure 3B, responses against the N-terminal region of MELOE-1 (2–21) were detected in 6/7 donors, with rather low frequencies (from 1 to 5% of positive microcultures), except in HD28 healthy donor, who exhibited 54% of positive microcultures. These microcultures contained between 0.7 to 6.4% of TNFα producing CD4 T cells (Figure 3C). The region 11–30 exhibited the ability to stimulate the growth of specific CD4 T cells in each tested donor (from 2 to 45% of positive microcultures containing between 0.6 to 23% of TNF-α producing CD4 T cells), and with high frequencies in three donors (HD17, HD24 and HD28). This suggested the presence, in this peptide, of at least one promiscuous epitope able to bind to various HLA contexts. On the contrary, the central region 18–37, containing an already described DR11-restricted epitope (24–37) located just at the end of this 20-mer peptide [23], was recognized by microcultures deriving from only one healthy donor (HD9, expressing the DR11 element). In this donor, we detected 4% of positive microcultures, containing between 1 and 3.8% of TNF-α producing CD4 T cells (Figure 3B and C). Finally, the C-terminal region (26–46), containing an already described DQ6-restricted epitope [23], was recognized by MELOE-1-stimulated microcultures from 4 out of 7 donors (only one expressing the DQ6 element), with frequencies ranging from 2 to 10% of microcultures containing between 0.8 and 17% of TNF-α producing CD4 T cells.

### Frequency, Distribution and Th Profile of MELOE-1 Specific CD4 Responses in Melanoma Patient’s PBMC Stimulated with MELOE-1 Antigen

In order to confirm the immunoprevalence of each MELOE-1 region in melanoma patients, and to characterize the Th type of CD4 specific T cells, we stimulated 10 melanoma patients PBMC with the MELOE-1 whole protein, and tested the reactivity of stimulated lymphocytes towards the regions: 2–21, 11–30, and 22–46. For this study, instead of challenging microcultures with the 18–37 peptide, appearing seldom recognized by MELOE-1 stimulated microcultures from healthy donors, we extended the N-terminus region of the 26–46 with four amino acids (22–46), in order to include our previously described HLA-DR11-restricted epitope (24–37). Indeed, we previously showed that CD4 T cells specific for MELOE-1_24–37_ epitope were efficiently induced by 22–46 peptide stimulation [23].

**Figure 4:**
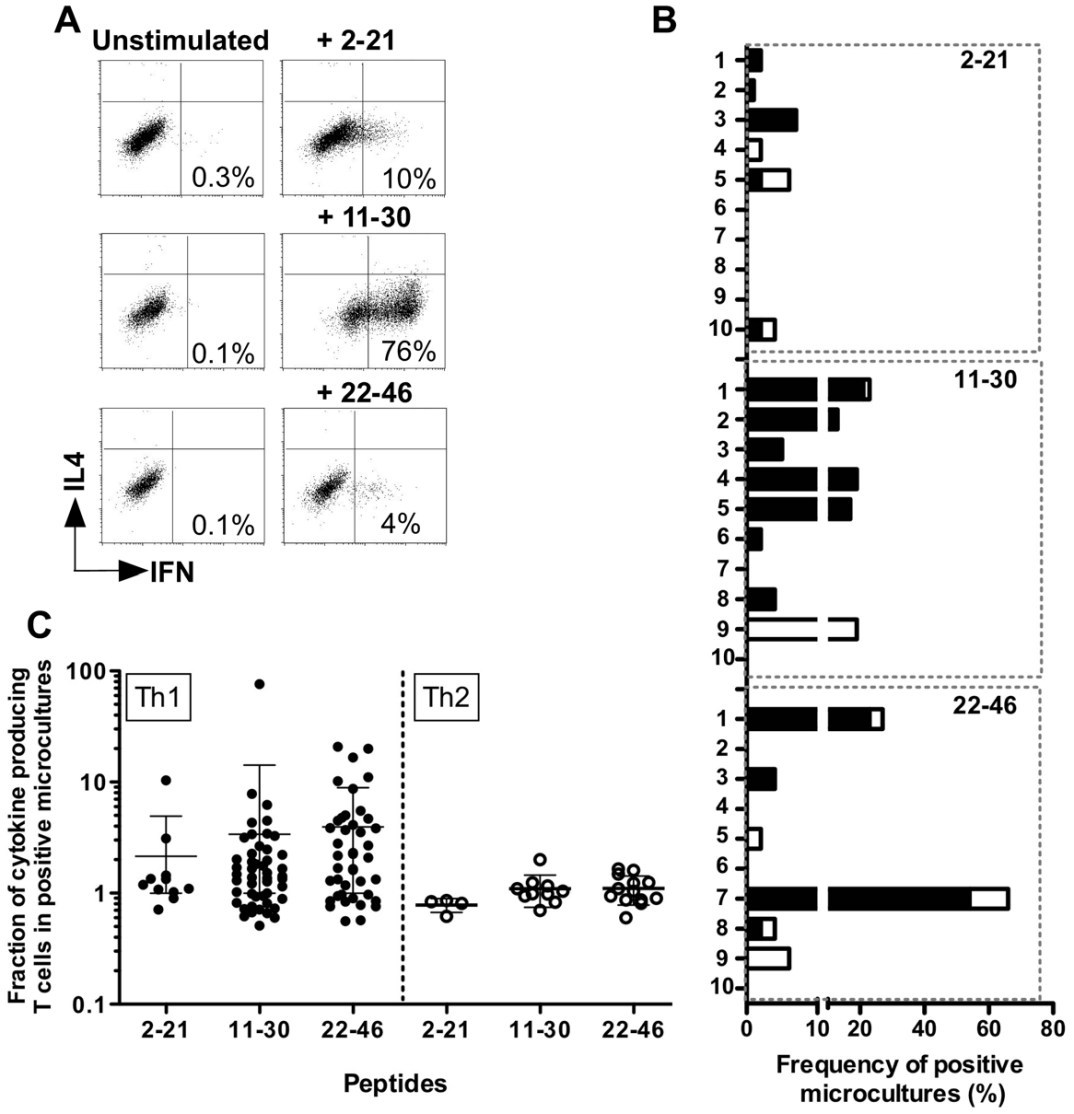
MELOE-1 specific responses in melanoma patients. (A) Dot plot illustrating peptide-specific Th1 responses in MELOE-1 stimulated microcultures from Pt≠3. Fourteen days after PBMC stimulation with MELOE-1 whole polypeptide (2–46), microcultures were re-stimulated with each indicated peptide during 5 h. Specificity was then assessed by a triple labelling IFN-γ/IL-4/CD4. Percentages indicate the fraction of IFN-γ producing T cells, among CD4 T cells. (B) Frequency of microcultures containing IFN-γ (black bars) or IL-4 (white bars) producing CD4 T cells specific for MELOE-1 overlapping peptides. A microculture was considered as positive when the fraction of cytokine producing CD4 T cells was 2-fold higher upon peptide stimulation than in unstimulated cultures. (C) Percentages of cytokine producing CD4 T cells in positive microcultures from all patients. IFN-γ (left panel) and IL4 (right panel) production was assessed by a triple labelling IFN-γ -IL4-CD4, after peptide re-stimulation.

Figure 4A illustrates specific responses of CD4 T cells derived from Pt≠3-stimulated microcultures, exhibiting specific Th1 CD4 responses (IFN-γ production) against each of these overlapping peptides.

**Figure 5:**
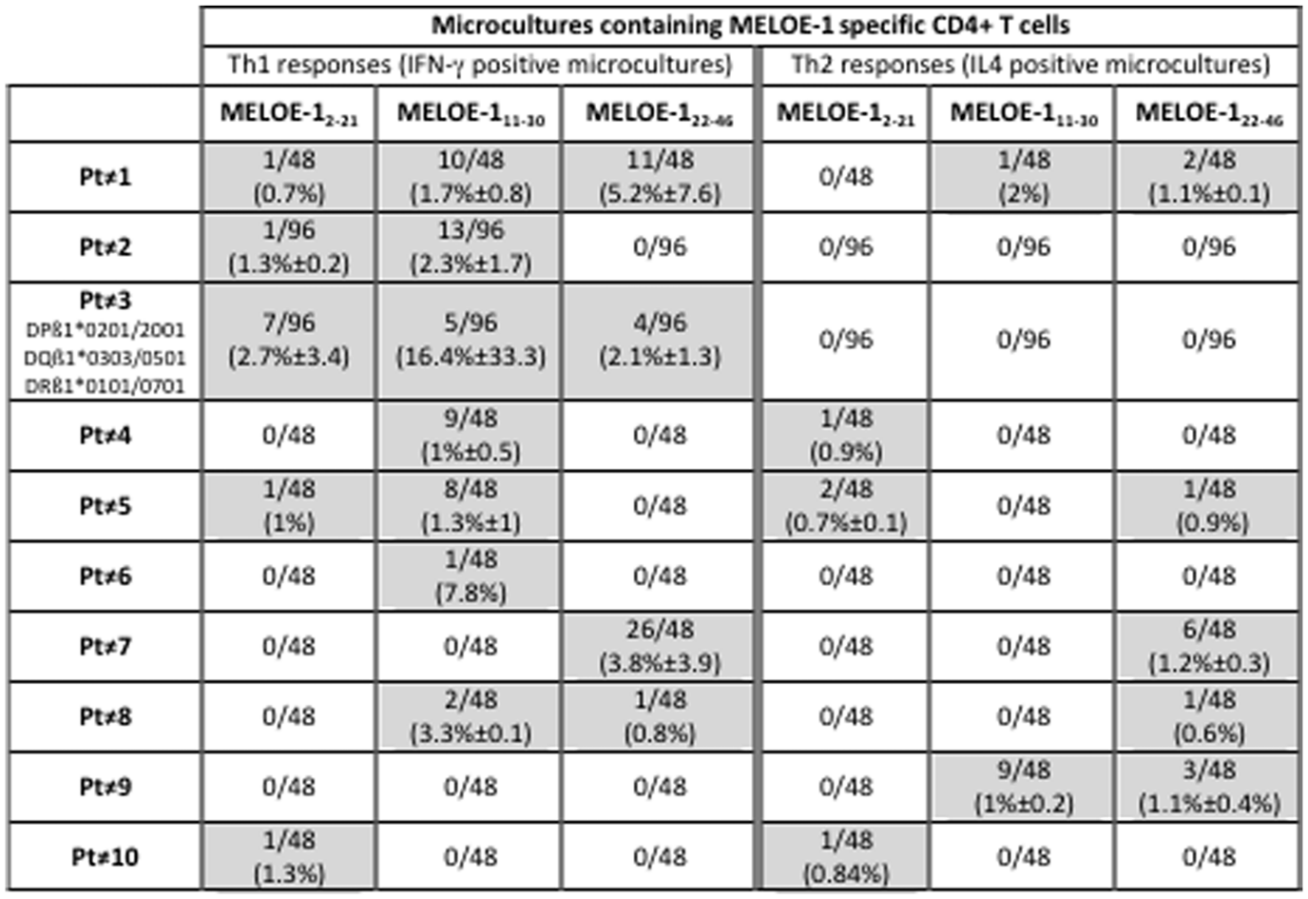
Assessment of MELOE-1 CD4 T cell responses in PBMC from melanoma patients. PBMC from melanoma patients were stimulated with 10 µM of MELOE-1. After 14 days, the presence of CD4 T cells specific for the different regions of MELOE-1 was assessed by re-stimulating cells with MELOE-1_2–21_, MELOE-1_11–30_ and MELOE-1_22–46_ peptides, followed by CD4/IFN-γ double staining for the detection of Th1 responses, and by CD4/IL4 double staining for Th2 responses. Between brackets is indicated the mean % of cytokine producing CD4 T cells, in positive microcultures.

On the basis of IFN-γ secretion, we documented the growth of MELOE-1 specific Th1 CD4 T cells in microcultures from all patients but one, and to a lower extent Th2 CD4 responses, assessed by IL-4 production, in 7/10 patients (Figures 4 and 5). Of note, the only patient exhibiting the highest frequency of Th2 responses (Pt≠9) was the only patient who did not exhibited MELOE-1 specific Th1 responses. As for healthy donors, the global frequency of MELOE-1 specific CD4 T cells can be roughly estimated and ranged from 2.5×10^−7^ to up to 6.5×10^−6^ among CD4 T cells from melanoma patients.

As observed for healthy donors, the central region of MELOE-1 (11–30) was frequently recognized by MELOE-1 stimulated microcultures. Indeed, we detected MELOE-1_11–30_ specific responses from 8/10 patients. These responses were mainly Th1 responses (Figure 4B, black bars) detected in 7 patients, with frequencies of positive microcultures ranging from 2% to 21%. Overall, positive these microcultures contained between 0.5 to 76% of IFN-γ producing CD4 T cells (Figure 4C, left panel). In contrast, microcultures containing Th2 CD4 T cells specific for this region were detected in only two patients (Pt≠1 and ≠9), with frequencies of 2 and 19% (Figure 4B, white bars). In these microcultures, fractions of IL-4 producing T cells were much lower, never exceeding 2% of CD4 T cells (Figure 4C, right panel).

Th1 CD4 T cells specific for MELOE-1_2–21_ and MELOE-1_22–46_ were detected respectively in 5 and 4 patients, with frequencies of positive microcultures ranging from 1 to 7% and 2 to 54% (Figure 4B). The fraction of IFN-γ producing T cells in positive microcultures ranged from 0.7 to 10.4% of CD4 T cells for MELOE-1_2–21_, and from 0.6 to 21% for MELOE-1_22–46_ (Figure 4C, left panel). Again, Th2 CD4 T cells specific for MELOE-1_2–21_ were less frequent, both in terms of frequency (2 to 4% of positive microcultures from 3/10 patients), and in terms of fraction of IL-4 producing T cells in positive microcultures, always below 2% (Figure 4C right panel). MELOE-1_22–46_ specific CD4 Th2 lymphocytes were more frequently induced (2 to 12% of positive microcultures from 5/10 patients), but the fraction of IL-4 producing T cells remained low (from 0.6 to 1.6% of CD4 T cells).

In summary, stimulation of patient’s PBMC with MELOE-1 preferentially induced Th1 CD4 T lymphocytes specific for diverse epitopes located all along the protein sequence.

### Production and Characterization of CD4 T cell Clones Specific for the Different Regions of MELOE-1

**Figure 6:**
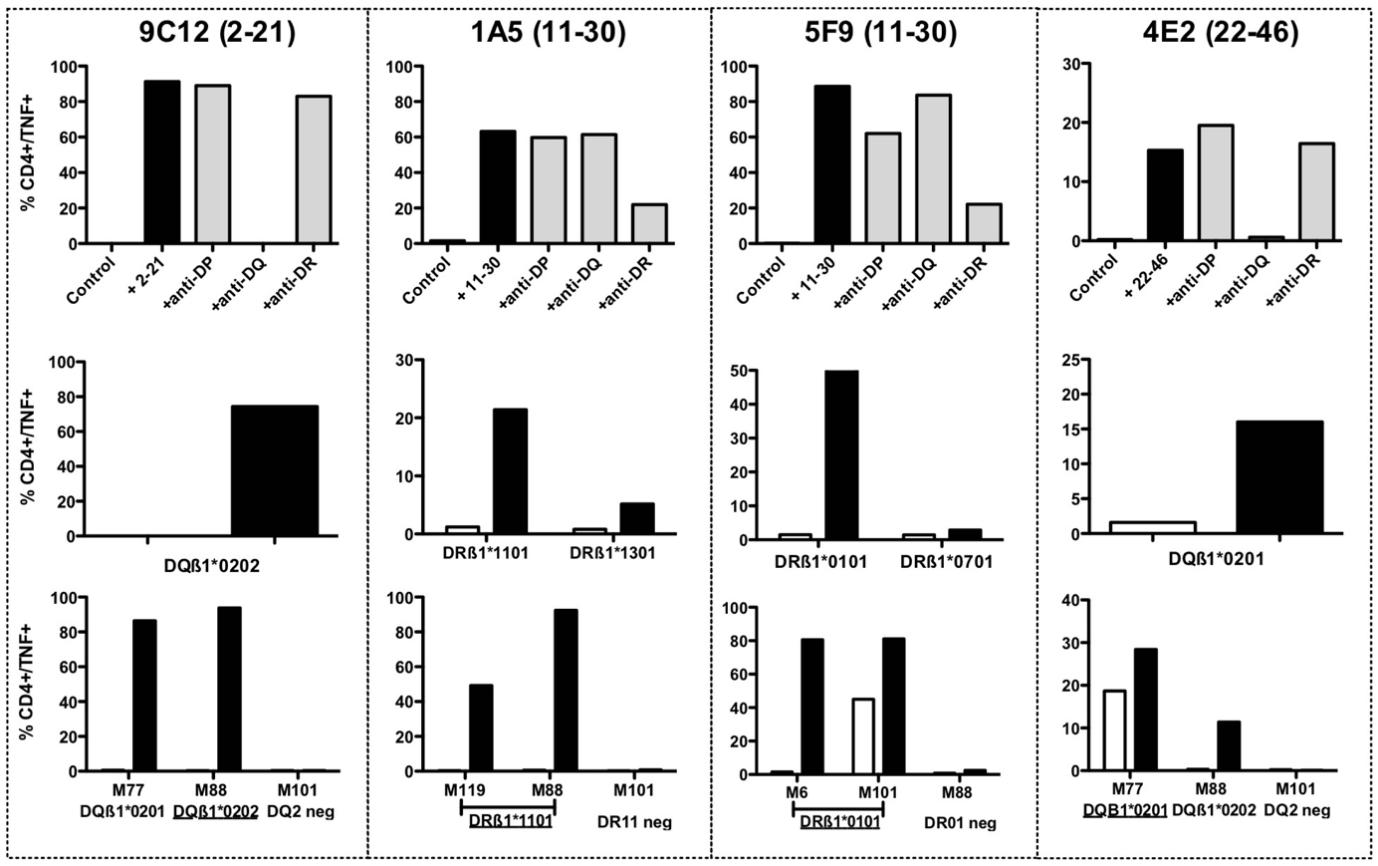
HLA-restricting element of MELOE-1 specific T cell clones and reactivity against HLA-matched melanoma cell lines. Upper panel: the HLA restriction of MELOE-1 specific T cell clones was assessed by intracellular TNF labelling, using anti-HLA blocking antibodies. T cell clones were stimulated either with peptide alone (10 µM) in an autopresentation assay and in presence or not of blocking antibodies at a concentration of 12.5 µg/mL. Middle panel: HLA restriction was confirmed with HLA-matched B-EBV cell lines unloaded (white bars) or pulsed (black bars) 2 h with the cognate peptide, at an effector/ratio of 1/2. Lower panel: reactivity of each T cell clone against HLA-class II expressing melanoma cells (ratio 1/2) was assessed by intracellular TNF labelling, in presence (black bars) or not (white bars) of exogenous peptide.

In order to formally characterize the recognized epitopes, we derived CD4 T cell clones specific for each region of MELOE-1 by limiting dilution, from microcultures of healthy donors or melanoma patients, containing at least 0.5% of specific CD4 T cells. We successfully derived CD4 specific T cell clones from HD17, HD22, HD25 and Pt≠3 microcultures, which were reactive against MELOE-1_2–21_ (HD22), MELOE-1_11–30_ (HD17 and Pt≠3) and MELOE-1_26–46_ (HD25). From each cloning experiment, we obtained between one and ten reactive CD4 T cell clones, that turned out to be the same clonotype after CDR3ß sequencing (Table 1 and data not shown). A single CD4 T cell clone for each specificity was used for further experiments. The HLA-restriction was determined for each T cell clone, first by using HLA-class II blocking monoclonal antibodies (Figure 6, upper panel), and further by testing the recognition of HLA-matched B-EBV cell lines loaded with each recognized long peptide (Figure 6 middle panel). The two T cell clones named 9C12 and 4E2, derived from HD22 and HD25 and specific for MELOE-1_2–21_ and MELOE-1_26–46_ were restricted by the HLA-DQß1*0202 and the DQß1*0201 molecules respectively. As these two donors were homozygous for the HLA-DQ locus, a single HLA-DQ matched B-EBV cell line was tested to confirm the HLA restriction of these two CD4 T cell clones. As shown on figure 6 (upper panel), the two other T cell clones 1A5 and 5F9 recognized the 11–30 region of MELOE-1, in a HLA-DR context. The use of HLA-matched B-EBV cell lines allowed to precise that 1A5 T cell clone was restricted by the HLA-DRß1*1101 molecule and the 5F9 T cell clone by the HLA-DRß1*0101 molecule.

**Table 1:** TCR characterization and cytokine profile of MELOE-1 specific CD4 T cell clones.

MELOE-1_2–21_ specific CD4 T cell clone (9C12- DQß1*0202)		
*CDR3 beta chain*		*Cytokine profile* a
V beta chain	Vß2.1	TNF^ high^
CDR3 beta	CSA SPDTHWGTDTQ YFG	IFN ^high^
J beta chain	Jß 2.3	IL2 ^high^
		GM-CSF^ high^
		IL4 ^high^
		IL5 ^low^
		IL13 ^high^
		IL10 ^neg^
MELOE-1_11–30_ specific CD4 T cell clone (1A5–DRß1*1101)		
V beta chain	Vß5.1	TNF^ high^
CDR3 beta	CAS SSAGGNSGNTI YFG	IFN ^high^
J beta chain	Jß 1.3	IL2 ^high^
		GM-CSF^ high^
		IL4 ^low^
		IL5 ^neg^
		IL13 ^neg^
		IL10 ^neg^
MELOE-1_11–30_ specific CD4 T cell clone (5F9-DRß1*0101)		
V beta chain	Vß8.2	TNF^ high^
CDR3 beta	CAS SRTGGNYGY TFG	IFN ^high^
J beta chain	Jß1.2	IL2 ^low^
		GM-CSF^ high^
		IL4 ^high^
		IL5 ^neg^
		IL13 ^high^
		IL10 ^neg^
MELOE-1_26–46_ specific CD4 T cell clone (4E2- DQß1*0201)		
V beta chain	Vß2.1	TNF^ high^
CDR3 beta	CSA SGRRKFYEQ YFG	IFN ^high^
J beta chain	Jß 2.7	IL2 ^high^
		GM-CSF^ high^
		IL4 ^high^
		IL5 ^low^
		IL13 ^high^
		IL10 ^neg^

a CD4 T cell clones were stimulated for 5 hours in the presence of brefeldin A (10 µg/mL) either with the cognate peptide (10 µM) in an autopresentation assay. After 5 hours of stimulation, cells were stained with APC-conjugated anti-CD4 mAb, fixed with 4% paraformaldehyde, labeled with PE-conjugated anti-cytokine mAb and analyzed by flow cytometry. A “high” cytokine production was defined with a threshold of 20% of producing CD4 T cells, in response to the peptide.

We further tested the reactivity of these CD4 T cell clones against HLA-matched melanoma cell lines positive for *meloe* expression and expressing HLA-DQ and DR at the cell surface. All the T cell clones were reactive against HLA-matched melanoma cell lines when loaded with the cognate peptide (Figure 6, lower panel, black bars). The two DQ2-restricted T cell clones were reactive against peptide-loaded DQß1*0201 and 0202 melanoma cell lines. In absence of peptide, only the 4E2 DQ ß1*0201-restricted T cell clone was able to recognize unloaded M77 melanoma cell line (also DQß1*0201), but not the DQß1*0202 melanoma cell line, M88. Similarly, the DR1-restricted T cell clone 5F9 also recognized one of the DRß1*0101 melanoma cell line, in the absence of exogenous peptide (M101).

We also documented the Th profile of each T cell clone, upon stimulation with the cognate peptide and analysis of cytokine production. All the clones expressed Th1 cytokines (TNF-α, IFN-γ, IL2 and GM-CSF). On the contrary, these clones differ in their expression of Th2 cytokines. Indeed, the two DQ2 restricted T cell clones (9C12 and 4E2) and the DR1 restricted one (5F9) also strongly express two Th2 cytokines (IL4, and IL13), whereas the DR11-restricted T cell clone only weakly expressed IL4 (Table 1). None of the CD4 T cell expresses IL10 or IL5 at a significant level.

### Processing of the Recognized Epitopes from Autologous DC Loaded with MELOE-1 Antigen

**Figure 7:**
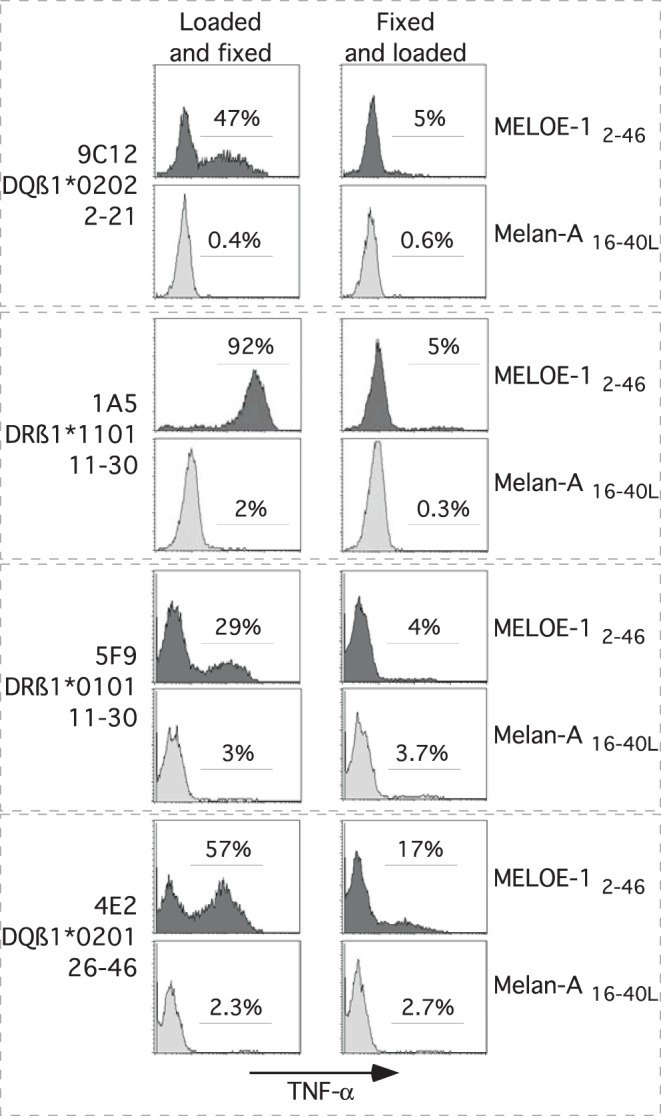
Class II epitopes are naturally processed from MELOE-1 whole antigen. Autologous DC, were loaded (before or after fixation) with MELOE-1_2–46_ (1 µM) or, as a negative control, with Melan-A_16–40L_ peptide (1 µM), and matured. T cell clones were then stimulated with DC at a ratio 1/1, during 5 h in presence of BFA. Reactivity was assessed by double staining TNF-α/CD3, and analyzed by flow cytometry. Histograms illustrated the percentage of TNF-α producing cells among CD3 positive T cells.

Initial PBMC stimulation was carried out with MELOE-1 whole antigen, and thus we assume that CD4 T cell responses were generated against peptides naturally processed by monocytes. Nonetheless, we could not formally exclude that the 14-day culture period did not artificially generate shorter class II epitopes that elicited CD4 T cell responses. Thus, it remained crucial to assess that all these new epitopes were naturally processed by autologous dendritic cells loaded with MELOE-1 whole protein, in serum-free medium. To this end, we loaded autologous iDC with 1 µM of MELOE-1 antigen in serum-free medium, in presence of maturating agents, and fixed these DC before stimulation of the CD4 specific T cell clones. In these conditions, the four T cell clones were reactive against MELOE-1 loaded autologous DC (Figure 7, left panel), whereas we could not detect any reactivity of CD4 T cell clones to DC loaded with an irrelevant long peptide, synthesized in the same conditions (Melan-A_16–40L_). As an additional control, we loaded autologous DC with MELOE-1 after DC fixation to formally exclude that T cell clone activation was not due to the exogenous loading of MELOE-1-derived shorter peptides, artificially generated by external degradation of the polypeptide. In this condition, responses of specific T cell clones were dramatically decreased, indicating that T cell clone activation was mainly due to the recognition of epitopes processed from MELOE-1 protein (Figure 7, right panel). Thus, the four new epitopes identified by PBMC stimulation, are naturally processed from MELOE-1 antigen.

### Characterization of the Minimal Recognized Epitopes

**Figure 8:**
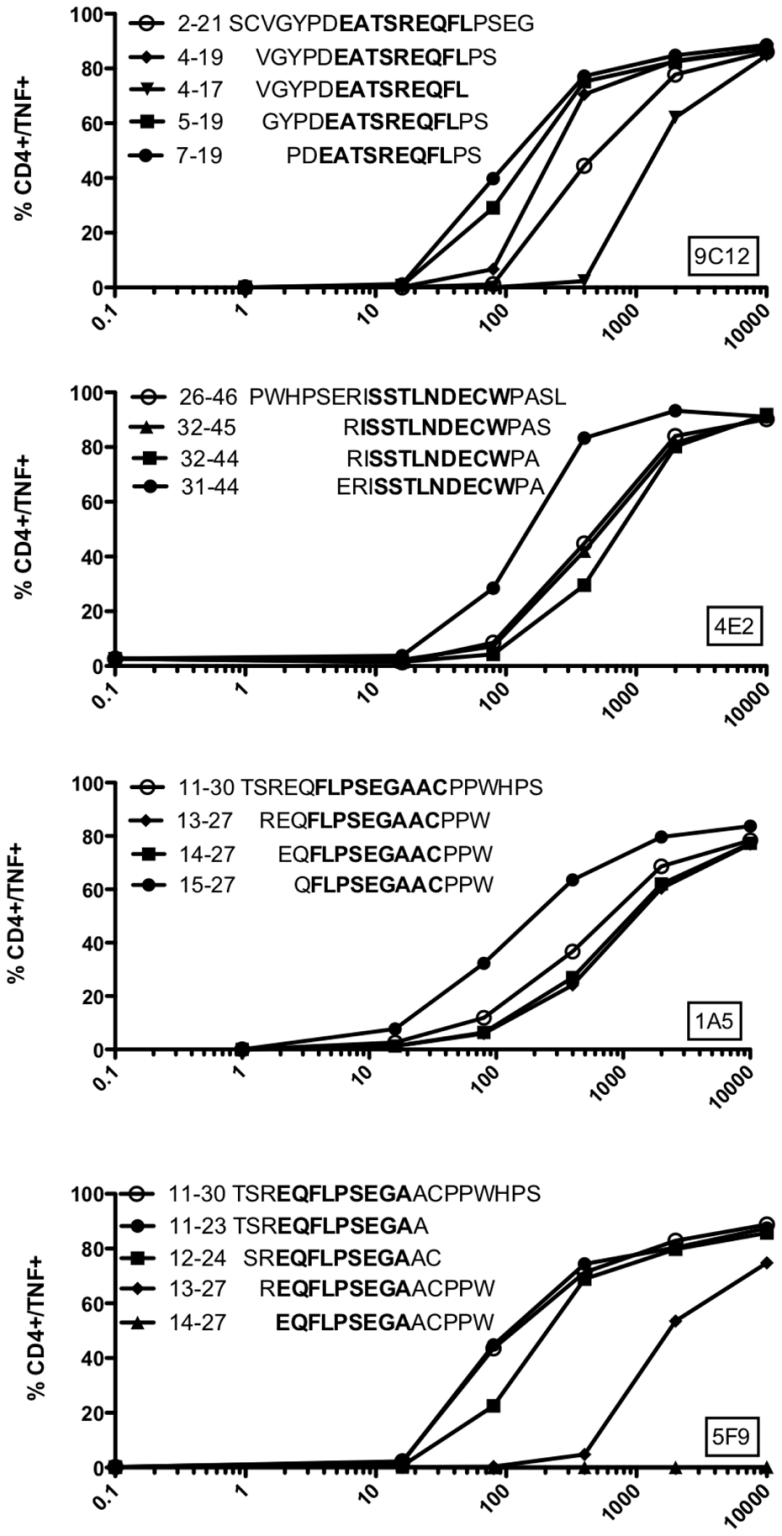
Minimal peptides recognized by MELOE-1 specific CD4 T cell clones. MELOE-1 specific CD4 T cell clones were incubated with various concentrations of the indicated peptides during 5 h in presence of BFA. TNFα production was assessed by intracellular labelling. The core peptide sequence is indicated in bold on each figure panel, and black circles illustrate the best fitting peptide.

Our T cell clones were reactive against 20-mer peptides that are probably not the exact peptides naturally processed. In order to formally identify the minimal recognized epitopes, we tested shorter peptides derived from each of the MELOE-1 recognized regions, chosen on the basis of the core peptide sequence supposed to be recognized by the T cell clones (deduced from the NetMHCIIpan website and indicated in bold on figure 8).

The DQ2-restricted 9C12 T cell clone better recognized three shorter peptides, the shortest one being MELOE-1_7–19_ (13-mer) recognized with an EC50 of 100 nM. The deletion of the two aa in C-term strongly reduces T cell clone recognition. The other DQ2-restricted T cell clone (4E2) better recognized a 14-mer peptide (31–44) with an EC50 of 100 nM, and also recognized to a lower extent the 32–44 epitope (Figure 8) previously described in the HLA-DQß1*0603 context [23]. Concerning the DR-restricted T cell clones, optimal shorter peptides were 13-mer peptides, MELOE-1_15–27_ for the DRß1*1101 restricted T cell clone 1A5 (EC50 = 100 nM), and MELOE-1_11–23_ or MELOE-1_12–24_ for the DRß1*0101 restricted one (Figure 8). Nonetheless all of these clones recognized a series of shortened peptides, thus we cannot formally assess that the shortest ones will be the exact peptides naturally presented on class II molecules.

## Discussion

Induction of CD4+ Th cells can be efficiently achieved through the use of SLP, as their length usually allows for the presentation of at least one Th epitope. Vaccination with peptides encompassing class II and class I HER-2neu derived epitopes has been tested in breast cancer patients and was shown to induce long-lived specific CD8 immune responses [28]. More recently, the induction of persistent CD4 responses has been documented after vaccination with a P53-derived long peptide in colorectal cancer patients [17]. From a clinical standpoint, the efficiency of long peptide vaccination has been documented through the observation of objective clinical responses in patients with vulvar intraepithelial neoplasia [18], [29]. In melanoma, long peptide vaccination has not been tested so far, and among the multiple melanoma antigens already described, MELOE-1 appears to be a relevant target because of the presence of a HLA-A2 restricted epitope that seems to be involved in melanoma immunosurveillance [21], of a large and reactive specific CD8+ repertoire in melanoma patients [22], and because of the length of this antigen (46 aa), compatible with its use as full-length antigen for peptide vaccination. We already documented the presence of class II epitopes in the vicinity of the HLA-A2 epitope, in the C-terminal region of this antigen [23]. The aim of the present study was to look for additional class II epitopes all along MELOE-1 sequence and to characterize the type of helper responses induced from MELOE-1-stimulated PBMC derived from melanoma patients.

In order to document the immunoprevalence of the different regions of MELOE-1 antigen, in terms of CD4 T cell stimulation, we stimulated healthy donor’s PBMC with MELOE-1 whole polypeptide, and showed that CD4 T cells specific for at least one region of this antigen were induced in each donor. This first result underlined the exceptional efficiency of this antigen to stimulated specific CD4 T cells. Indeed, such a high immunoprevalence has been observed only for few tumor antigens, such as NYESO-1 [30], [31], [32] and survivin [24]., eliciting CD4 specific responses in at least half tested patients.

This also suggested the existence of multiple class II epitopes all along the MELOE-1 sequence. This was further confirmed by the formal identification of four new epitopes located in various regions of the antigen: MELOE-1_7–19_, MELOE-1_11–23_, MELOE-1_15–27_ and MELOE-1_31–44_, respectively recognized in DQß1*0202, DRß1*0101, DRß1*1101 and DQß1*0201 contexts. Among these new epitopes, those located in the 11–30 region induced more frequently and more efficiently the growth of CD4 specific T cells compared to the other regions, suggesting a high immunoprevalence for this specific region. This suggested that these epitopes could be promiscuous epitopes able to bind with a sufficient affinity to one or various HLA-alleles.

In order to precisely characterize MELOE-1 specific CD4 T cells in melanoma patients, we documented the Th1 and Th2 profile of specific CD4 T cells induced after PBMC stimulation with MELOE-1 antigen. Indeed, among the various sub-populations of anti-tumour CD4^+^ T cells, CD4 Th1 cells are of particular interest through their ability to enhance the function of antigen presenting cells and to target the tumour stroma and angiogenesis through the secretion of TNF-α and IFN-γ [33]. Furthermore, IFN-γ secreting Th1 cells are able to induce memory cytotoxic responses and strong cellular immunity. On the contrary, Th2 cells are thought to suppress cellular immunity through IL4 and IL10 production. For the screening of responses induced in melanoma patients PBMC, we decided to extend the 26–46 peptide at the N-term end, so that MELOE-1_24–37_ epitope will be included in this new long peptide (22–46). Indeed, results obtained on healthy donors suggested that MELOE-1_24–37_ was the only epitope located in the 18–37 region, because specific responses were only detected in one HLA-DR11 donor. We show that stimulation of melanoma patients PBMC with MELOE-1 whole antigen induces the growth of Th1 CD4 T cells specific of the various regions of the antigen in all melanoma patients but one (Figure 4B and 5). Fractions of specific Th1 cells among positive microcultures ranged from 0.5 to 76%, underlying the efficiency of MELOE-1 stimulation (Figure 4C). On the contrary, Th2 responses could be detected in 7/10 patients, but the fraction of specific T cells in positive microcultures was rather low, never exceeding 2% of CD4 T cells. It is interesting to note that the only patient that did not exhibit Th1 responses against MELOE-1 derived peptide (Pt≠9), showed the highest frequency of Th2 responses that were specific for MELOE-1_11–30_ and MELOE-1_22–46_ regions. This suggested that, in this peculiar patient, Th2 responses could have impaired the development of Th1 specific T cells. Overall, MELOE-1 specific CD4 T cells were not more frequently detected in melanoma patients’ PBMC than in healthy donors’ PBMC. This would suggest that overexpression of *meloe* messenger mRNA in tumor cells did not result in the expansion of specific CD4 T cells *in vivo* in melanoma patients. However, considering that we previously showed that MELOE-1 specific CD8 T cells were enriched within melanoma TIL populations with no increase of these specific T cells in the periphery, a similar situation may occur with specific CD4 responses that could be exclusively expanded in the tumor site but not in the periphery.

As observed in healthy donors, the central region 11–30 was frequently recognized by MELOE-1 stimulated PBMC, as well as the C-term extended region (Figure 4B). Overall, the two main informations obtained from the analysis of melanoma patients are the strong immunoprevalence of MELOE-1 antigen, especially of the central and C-term regions, and the ability of this melanoma antigen to induce specific CD4 Th1 lymphocytes, favourable to persistent anti-tumour responses.

In order to formally characterize some of the recognized class II peptides, we derived CD4 T cells clones specific for the epitopes located within the different regions of MELOE-1 (from 3 healthy donors, and from one melanoma patient). We could thus characterize the Th profile of these clones, and their functional properties. As expected, all these clones exhibited a phenotype of experienced T cells: CD45RO^high^, CD28^high/low^, CD27^neg^ (data not shown). Considering the dominant Th1 profile assessed by IFN-γ production in MELOE-1 stimulated microcultures from melanoma patients, we expected to derive Th1 CD4 specific T cell clones. Indeed, all the T cell clones expressed Th1 cytokines (TNF-α, IFN-γ, IL-2 and GM-CSF) but 3 out of 4 T cell clones also expressed high levels of IL-4 and IL-13, known as Th2 cytokines. Nonetheless, none of these clones expressed IL-10 or IL-5 at a significant level. The production of IL-4 and IL-13 by CD4 T cell clones expressing Th1 cytokines was already described in our previous work [23]. Accordingly, a recent study reported a mixed Th1/Th2 profile for tumour specific T cell clones derived from patients undergoing peptide vaccination treatments [34]. In this study, the authors underline that the dichotomy Th1/Th2 is not easily applicable to tumour specific T cells in humans, and that the ratio IFN-γ/IL-10 could be an indicator of responses associated to long-term survivors, rather than the delineation of clear Th1 or Th2 profiles. In our study MELOE-1 derived specific CD4 T cell clones exhibited a high ratio IFN-γ/IL10, thus favourable to the development and the persistence of anti-tumour responses. It is strongly improbable that these T cell clones were T reg clones because of their high proliferative capacity, and of their ability to secrete IL2. Indeed, even if some tumor antigen specific CD4 Treg clones were also found to secrete a diverse panel of cytokines such as IFN-γ, GM-CSF, TNF and IL5 or IL4 and IL10, *in vitro*, none of them was able to secrete IL2 [35], [36], [37].

We further documented the reactivity of specific CD4 T cell clones to HLA-matched MELOE-1 expressing melanoma cell lines, in presence or absence of exogenous peptide. Spontaneous recognition of tumour cells by CD4 T cell clones depends on the nature of the antigen and on its subcellular localization. We previously reported the absence of recognition of HLA-matched melanoma cell lines by two CD4 T cell clones specific for the C-term region of the antigen [23], that seemed consistent with the fact that MELOE-1 has no signal peptide, nor endosomal targeting sequence. In this study, we documented the spontaneous recognition of two melanoma cell lines by two CD4 T cell clones specific for the central region 11–30 in the DR1 context (5F9) and the C-term region in the DQ2 context (4E2) (Figure 6). Thus some peptides, derived from MELOE-1 antigen, could be presented by class II molecules at the tumour cell surface, and other peptides could not. This dichotomy between class II peptides derived from a same tumour antigen has already been described for the MAGE-A3 antigen [38]. This suggest that some peptides derived from MELOE-1 could be able to reach endosomal compartments, and thus to be loaded on HLA class II molecules of some melanoma cell lines. The elucidation of the mechanisms leading to such disparities between class II tumour-derived epitopes could be of interest for future vaccination strategies.

As all of these new epitopes were not presented on melanoma cells, a crucial point to document was their ability to be processed by autologous DC from MELOE-1 whole protein. We thus loaded autologous DC with MELOE-1 whole protein and measure the activation of each specific CD4 T cell clone. Alternatively, to ensure that T cell clone activation was not due to the external loading of shortened peptides (derived from MELOE-1 degradation), we fixed DC before MELOE-1 loading. We observed a strong activation of all CD4 T cell clones, only when MELOE-1 was loaded on unfixed DC, showing that all the new class II epitopes were effectively processed by MELOE-1 loaded DC. This result supports the immunological relevance of these new class II epitopes.

In conclusion, we showed that MELOE-1 antigen contains various class II epitopes, located all along the sequence, which are naturally processed from MELOE-1 whole protein, and able to be recognized in multiple HLA class II contexts. Furthermore, stimulation of patients’ PBMC with MELOE-1 antigen preferentially induced the growth of CD4 Th1 lymphocytes specific for these epitopes. Therefore, in a vaccination perspective, the use of MELOE-1 whole protein appears relevant, as Th1 responses, supposed to favour anti-tumour responses, could be induced from a majority of melanoma patients.
